# The diagnostic accuracy of Th1 (IFN-γ, TNF-α, and IL-2) and Th2 (IL-4, IL-6 and IL-10) cytokines response in AFB microscopy smear negative PTB- HIV co-infected patients

**DOI:** 10.1038/s41598-019-39048-x

**Published:** 2019-02-27

**Authors:** Job Kisuya, Alex Chemtai, Evans Raballah, Alfred Keter, Collins Ouma

**Affiliations:** 1 Department of Biomedical Science and Technology, Maseno University, Private Bag, Maseno, Kenya; 2 Academic Model for Providing Access to Healthcare (AMPATH), P.O Box 4606-30100, Eldoret, Kenya; 3 Department of Immunology, Moi University, P.O. Box 4606-30100, Eldoret, Kenya; 4 Department of Medical Laboratory Sciences, Masinde Muliro University of Science and Technology, P.O. Box 190-50100, Kakamega, Kenya; 5 Centre for Global Health Research/Kenya Medical Research Institute, P.O. Box 1578-40100, Kisumu, Kenya; 6 Ideal Research Centre, P.O. Box 7244-40123, Kisumu, Kenya; 7 Center for Global Health, Department of Internal Medicine, University of New Mexico Health Sciences Center, Albuquerque, NM USA

## Abstract

Acid Fast Bacilli (AFB) microscopy smear remains the most widely used laboratory diagnostic technique for Pulmonary Tuberculosis (PTB) in low-and-middle income countries. Although it is highly specific, the sensitivity varies between 20–80% in immune-competent people, with only 50% case detection among HIV/TB co-infected patients, hence the need to determine the diagnostic accuracy of Th1 and Th2 cytokine response in AFB microscopy smear negative PTB-HIV co-infected patients. A total of 86 participants were recruited; 70 (81.4%) AFB microscopy smear negative and 16 (18.6%) AFB microscopy smear positive. The AFB microscopy smear negative samples were then cultured using Lowenstein Jensen Medium with 46 being culture-negative and 24 being culture-positive. Blood samples were also collected, cultured using QFT-GIT and the supernatant (plasma) harvested to evaluate cytokine profiles using Enzyme-Linked Immunosorbent Assay. IFN-γ (*P* < 0.001), TNF-α (*P* = 0.004), IL-2 (*P* = 0.004) and IL-4 (*P* = 0.009) median levels were elevated in PTB culture-positive (AFB microscopy smear negative) as compared to PTB culture-negative (AFB microscopy smear negative) participants. Finally, when Th1 cytokines (IFN-γ, TNF-α and IL-2), Th2 cytokines (IL-6 and IL-10) and T cells were included in the logistic regression fit for PTB outcome, the predictive power of discriminating between those who were AFB smear negative in the diagnosis of PTB was good with cross validated area under the curve (AUC) being 0.87 (95% CI: 0.78, 0.96). This study provides evidence for the ability of Th1 and Th2 cytokines to determine PTB status in AFB microscopy smear negative patients co-infected with HIV.

## Introduction

Tuberculosis (TB) is among the main HIV and AIDS defining illnesses^1^. In 2016 there were an estimated 10.4 million new cases of TB worldwide, with 1.2 million people living with HIV developing TB. TB is the leading cause of death among people living with HIV, accounting for 400,000 deaths in HIV-associated TB. Africa accounted for 75% of all deaths^2^. Kenya is among countries with the highest annual number of TB cases, with an estimated incidence of 348 per 100,000 population and 36,000 incident TB cases occurring among persons living with HIV infection^2^.

*Mycobacterium tuberculosis* (MTB) complex is the causative agent of TB, one of the oldest diseases known to affect humans. Although all MTB complex members are obligate pathogens and cause TB, they exhibit distinct phenotypic characteristics and host range. MTB is a rod-shaped, non-spore-forming, thin aerobic bacterium measuring about 0.5 µm by 3 µm and it does not stain readily and are often neutral on Gram’s staining. However, once stained, the bacilli cannot be decolorized by acid alcohol, a characteristic justifying their classification as acid-fast bacilli (AFB)^3^.

AFB microscopy smear is the most widely used laboratory technique for pulmonary tuberculosis (PTB) diagnosis in low to middle income countries, such as Kenya. Although it is highly specific, its sensitivity has been found to vary between 20% and 80%^4^. Furthermore, this conventional AFB staining method accounts for only 50% case detection among HIV/TB co-infected patients. However, the challenges related to this method of detection of MTB in HIV and AIDS still remain largely unresolved due to the nature of PTB disease in HIV-infected person. Sputum smear microscopy, which is the most commonly available diagnostic method in resource constrained countries, is often rendered false negative in HIV individuals^5^.

TB presentation in HIV-infected individuals with well-preserved immunity is similar to that in immune-competent individuals without HIV. The progression of immunodeficiency, attenuation of host tissue-damaging responses and failure of MTB containment result in an increased likelihood of atypical presentation, and greater proportions of extra-pulmonary and disseminated disease^5^. Sputum samples should be examined even if the chest radiograph appears normal. This is because pulmonary cavitations are less common in HIV-positive patients, leading to significantly reduced sensitivity of sputum microscopy for AFB. Therefore, it is necessary to further examine the sputum samples as culture still remains the mainstay of diagnosis in our setup, with limitation of being very expensive and taking a longer period to get results.

Molecular assays show promise in the rapid diagnosis of smear-negative disease with high sensitivity^6^. One of these tests is the Interferon Gamma Release Assay (IGRA). The challenge with IGRA is that it is dependent on T cell functionality and it is likely that such an assay might not work accurately in immune-challenged individuals, with infections such as HIV. However, the MTB-specific antigens present in IGRA test have great potential to stimulate T-cells to produce multiple cytokines, which could prove beneficial in the diagnosis of PTB among HIV co-infected patients. In addition studies have demonstrated the inability of interferon gamma alone to accurately diagnose TB in HIV-infected patients^7,8^. Furthermore, other studies have shown that IGRA as currently constituted, cannot be used alone to rule out active TB in HIV individuals, because it doesn’t add value to the discriminating ability of TB screening clinical algorithms^9,10^.

Previous studies have shown the ability of Th1 and Th2 cytokines in discriminating between PTB culture-positive and -negative^11,12^. Hence, the present study was designed to determine diagnostic accuracy of Th1 (IFN-γ, TNF-α, and IL-2) and Th2 (IL-4, IL6 and IL-10) cytokine response in AFB microscopy smear negative PTB-HIV co-infected patients.

## Methods and Materials

### Study setting

The study was carried out at Academic Model for Providing Access to Healthcare (AMPATH) based in Western Kenya. AMPATH is a collaboration among Moi University’s School of Medicine (MuSoM), Moi Teaching and Referral Hospital (MTRH) and a consortium of North American led by Indiana University^13^. Previously, AMPATH mainly focused on the delivery of HIV care, but over the past several years, it has broadened its mandate to include primary health care and chronic disease management, including prevention, diagnosis and treatment services for TB.

### Study Population

The study population was a prospective cohort of individuals newly diagnosed with pulmonary TB and HIV, and attending clinics at MTRH and AMPATH. The inclusion criteria were patients aged over 18 years, without prior history of TB or any TB-relevant clinical or radiological findings or relapse. Patients who had one or more TB specific symptoms and signs based on 2013 WHO guidelines for management of TB and leprosy in Kenya, were eligible for the study^14^. Recruited study participants were those who voluntarily accepted to be tested for HIV and were naïve for highly active antiretroviral therapy (HAART) and anti-TB treatment.

Patients who were pregnant, diabetic, or otherwise immunologically-challenged or harboring an autoimmune disease were excluded from the study. The above-mentioned diseases and medical conditions are associated with the modification of immune responses, and therefore could alter the study’s findings and conclusions.

In order to attain a power of 80% and to detect an effect size of 0.65, where the estimated IFN-γ mean for the culture negative is 12.99 ± 5.7 pg/mL, and estimated IFN-γ mean for the culture positive is 48.69 ± 28.78^15^, we needed a minimum sample size of 72 (i.e. 36 culture-positive and 36 culture-negative). Following this criteria, however, the total sample size for our study was 86 i.e. 40 in the culture-positive group and the 46 in the culture-negative group. In all our procedures (from handling patients to laboratory procedures), measures were taken to adequately control biohazards, particularly with respect to protection of individuals and the environment.

### Laboratory procedures

#### Sputum Smears

Ziehl-Neelsen Direct Microscopy: All Sputum samples collected from the study participants, were smeared, stained by Ziehl-Neelsen (ZN), and observed for the presence or absence of acid-fast bacilli under a light microscope with a 100X objective lens under oil immersion.

Culturing-(Lowenstein-Jensen Medium) and ZN Staining: The AFB smear negative samples were further cultured using Lowenstein-Jensen medium. Positive cultures were confirmed by ZN staining and species determined by capilia (FIND and Tauns Co. Ltd) according to the manufacturer’s instructions. A definite TB case was defined as a positive culture confirmed by speciation. Culture-positive AFB microcopy (smear negative) were considered to have PTB and culture-negative AFB microscopy (smear negative) were considered not to have PTB.

Immunophenotyping: Whole blood samples were also collected into 4 mL EDTA-containing vacutainer tubes (Becton Dickinson, Franklin Lakes, NJ, USA). Two combinations of 4 monoclonal antibodies were used (anti-CD3/CD8/CD45/CD4 and anti-CD3/CD16 + 56/CD45/CD19 Catalogue # 342416) and TruCOUNT tubes (Becton Dickinson, NJ, USA). About 20 μL of MultiTEST CD3FITC/CD8PE/CD45PERCP/CD4APC (Becton Dickson, NJ, USA, Catalogue # 342417) reagent was pipetted into the bottom of one tube while another 20 μL of MultiTEST CD3FITC/CD16 + CD56PE/CD45PERCP/CD19APC into the bottom of a second tube. About 50 μL of well-mixed, anti-coagulated whole blood sample was pipetted into the bottom of each tube. The tubes were capped and vortex gently to mix, then incubated for 15 minutes in the dark at room temperature (20–25 °C). Following this step, 450 μL of the Lysing Solution (Becton Dickson, NJ, USA, Catalogue # 349202) was added into each tube. The tubes were again capped and vortexed gently to mix and incubated for 15 minutes in the dark at room temperature (20–25 °C). Determination of lymphocyte subsets was performed using a FACSCalibur flow cytometer (Becton Dickinson Immunocytometry Systems, San Jose, CA, USA) on single technology platform. Four-color staining was performed, and at least 10^5^ cells were analyzed on a FACSCalibur equipped with a two-laser system (488- and 630-nm wavelength, respectively). Lymphocyte subsets were acquired using MultiSET software (Becton Dickinson, NJ, USA) and analyzed using Flowjo (Tristar Inc, Herzenberg, Stanford, US). The absolute count of the cell population (A) was calculated using the equation below as per BD Multitest kit manufacturer instruction:

A = X/Y x N/V, where:

X is the number of positive cell events

Y is the number of bead events

N is the number of beads per test

V is the sample volume (50 µL)

Interferon Gamma Release Assay (IGRA): Blood was cultured using the Quantiferon TB Gold-in-Tube-test (QFT-GIT) (Cellestis, Qiagen GmBH, Carnegie, Victoria, Australia, Cat #05900301) kit as per the manufacturer’s instructions. One mL of blood was placed into each of the three tubes that were pre-coated with either TB antigen, phytohemaglutinin for positive control or nil antigen for negative control. The tubes were then incubated for 16–24 h at 37 °C and the supernatant (plasma) were harvested after centrifugation, and were snap-frozen at −80 °C until use.

Measurement of Cytokines: The frozen aliquots of cultured QFT-GIT supernatant from the three tubes namely TB antigens, Control tube and Nil tubes from the each study participants, were thawed and the cytokine levels for IL-2, IL-4, IL-6, IL-8, IL-10, IL-12 (p70), TNF-α and IFN-γ in QFT-GIT supernatants were measured by standard sandwich ELISA technique using GenWays Biotech Kit (Greenways Biotech Inc., San Diego, CA, USA; Catalogue #s GWB-ZZD007, GWB-ZZD002, GWB-ZZD006, GWB-ZZD013, GWBZZD005, GWBZZD009, GWBZZD003 and GWB-ZZD004) as per the manufacturer’s instructions. In brief, the test cytokine-specific monoclonal antibodies were pre-coated to the plate and the human-specific detection polyclonal antibodies were biotinylated. The QFT-GIT supernatant and biotinylated detection antibodies were added to the wells subsequently and followed by washing with 0.01 M Phosphate Buffered Solution (PBS). Avidin-Biotin-Peroxidase Complex was added and unbound conjugates were washed away with 0.01 M PBS. Horseradish peroxidase substrate (HRP) 3,3′,5,5′-tetramethylbenzidine (TMB) was used to visualize HRP enzymatic reaction. TMB was catalyzed by HRP to produce a blue color product that changed into yellow after adding an acidic stop solution. The Optical Density (OD) of each well was measured within 5 minutes of stopping the reaction using an ELISA microplate reader. Standard curves were plotted to determine the respective cytokines concentrations (as measured by OD).

Statistical Analysis: Data analysis was performed using software for statistical computation (R Core Team, 2016). Categorical variables such as gender were summarized as frequencies and their corresponding percentages. Continuous variables were assessed for Gaussian assumptions using Shapiro Wilks test and normal probability plots. Those that met the Gaussian assumptions were summarized as mean and the corresponding standard deviation (SD), while those that violated the assumptions were summarized as median and their corresponding interquartile range (IQR).

In order to estimate the amount of cytokines produced as a result of stimulation by *Mycobacterium tuberculosis* specific antigen present in QFT-GIT, we subtracted unstimulated cytokine levels in nil tubes from the stimulated cytokine levels in TB antigen tubes. The resultant cytokine levels were then used in the statistical analysis.

Means between two groups; culture-positive AFB microcopy (smear negative) and culture-negative AFB microscopy (smear negative) were compared using independent sample t-test. The medians were compared using two-sample Wilcoxon rank sum test. Pearson’s Chi-square test was used to compare the distribution of male and female participants across the culture and AFB microscopy smear status. In order to assess the predictive ability of Th1 and Th2 in the diagnosis of the PTB status (culture status used as the confirmed outcome), binary logistic regression models were fitted for Th1 [IFN-γ, TNF-α, IL-2 and IL-12(p70)] and Receiver Operating Characteristic (ROC) curves plotted. The area under the curve (AUC) was calculated to assess the predictive power of the model in giving us the correct diagnosis. Statistical significance was assessed at *P* ≤ 0.05.

The variables included in the models for the combined ROC curves were selected based on their association with the culture status findings. The Th1 cytokines that were significantly associated with the culture status were included in the model for predicting Th1 cytokines ROC curve. The Th2 cytokine that was significantly associated with the culture status was IL-4, although it was eliminated due to inherent multicollinearity measured using variance inflation factor that was > 5 units. The IL-6 and IL-10 were not associated with the culture status but were included since they improved the predictive power of the model. All models were assessed for multi-collinearity. There was no evidence of multi-collinearity since the variance inflation factor was 1 for all the cytokines included in the models.

In the analysis of the predictive power of the cytokines on PTB, the AUC and the corresponding 95% confidence intervals from cross validated data set, were included. Using the R packages “cvAUC” and “ROCR”, 10 validation folds were used to cross validate the AUC. For each validation fold, predicted values from trained and tested logistic regression fit, were generated. The sensitivity and specificity was derived from the ROC curve.

### Ethical Considerations

The study was approved (# 0001163) by Institutional Research and Ethics Committee (IREC), Moi Teaching and Referral Hospital, Kenya. Informed consent was obtained from all the study participants prior to recruitment into the study and all experiments were performed in accordance with relevant guidelines and regulations.

## Results

### Demographic characteristic of AFB microscopy smears status

This study enrolled a total of 86 participants who met the inclusion criteria. After subjecting the sputum to AFB smear microscopy, 70 (81.4%) were AFB microscopy smear negative and 16 (18.6%) were AFB microscopy smear positive. The AFB microscopy negatives were further cultured using Lowenstein Jensen Medium with 46 being culture-negative and 24 being culture-positive. The male to female ratio of study participants in the two groups were almost equally distributed as shown in Table 1. The mean age was 38.8 (SD = 12.3) years and 40.0 (SD = 11.1) years, respectively, for AFB microscopy smear negative and AFB microscopy smear positive. The demographic characteristics (age and gender) presented in this study were comparable between AFB microscopy smear-negative and -positive (Table 1).

**Table 1:** Demographic characteristic of relationship AFB microscopy smear status.

	N	AFB Smear Results		*P*-value
		Negative (N = 70)	Positive (N = 16)	
		Mean (SD) or n (%)	Mean (SD) or n (%)	
Age (years)	86	38.8 (12.3)	40.0 (11.1)	0.721^a^
**Gender**				
Male	86	35 (50.0%)	7 (43.8%)	0.652^b^
Female		35 (50.0%)	9 (56.2%)	

There was no difference in the demographic characteristics (age and gender) between those who were PTB culture-positive AFB microscopy smear negative and those who were PTB culture-negative AFB microscopy smear negative. Compared using ^a^independent sample t-test, and ^b^Pearson’s Chi-square test.

### Immunophenotypic Characteristic, Th1 and Th2 cytokines levels in PTB culture-negative and -positive AFB Microscopy smear negative status

A two-sample Wilcoxon rank sum test was performed to determine the relationship between immunophenotypic characteristic in PTB culture-negative and -positive AFB microscopy smear negative status. The median levels of CD4 and CD19 counts were elevated in PTB culture-positive AFB microscopy smear negative compared to PTB culture-negative AFB microscopy smear negative status, CD4 206.5 cells/µL (101.0, 315.5) vs 67.5 cells/µL (22.5, 192.5), *P* = *0*.*033* and CD19 96.5 cells/µL (29.5, 143.3) vs 46 cells/µL (15.5, 99.30), *P* = *0*.*048* (Table 2). Although the median levels of CD8 and CD16/CD56 were lower in PTB culture-negative AFB microscopy smear negative compared to PTB culture-positive AFB microscopy smear negative 473.0 cells/µL (157.8, 1166.0) vs 582.5 cells/µL (217.5, 945.5), *P* = *0*.*797* and 63.0 cells/µL (33.3, 113.5) vs 107.5 cells/µL (56.6, 152.3), *P* = *0*.*123*, respectively, they were comparable between the two groups (Table 2).

**Table 2:** Relationship bettween Immunophenotypic characteristics, Th1 and Th2 cytokines in PTB culture-negative and -positive of AFB microscopy smears negative status.

	N	Culture Results		*P*-value
		Negative (N = 46)	Positive (N = 24)	
		Median (IQR)	Median (IQR)	
CD8 cells/µL	70	473.0 (157.8, 1166.0)	582.5 (217.5, 945.5)	0.797
CD4 cells/µL	70	67.5 (22.5, 192.5)	206.5 (101.0, 315.5)	0.033
CD16/CD56 cells/µL	70	63.0 (33.3, 113.5)	107.5 (56.6, 152.3)	0.123
CD19 cells/µL	70	46.0 (15.5, 99.3)	96.5 (29.5, 143.3)	0.048
IFN-γ pg/mL	70	7.6 (5.6, 16.9)	70.6 (8.6, 202.7)	<0.001
TNF-α pg/mL	70	16.1 (14.4, 19.2)	19.7 (15.8, 22.9)	0.004
IL-2 pg/mL	70	15.3 (8.4, 26.9)	53.1 (14.1, 122.1)	0.004
IL-8 pg/mL	67	154.2 (50.1, 204.5)	118.1(73.8, 158.6)	0.174
IL-12p70 pg/mL	67	2.1 (0.7, 3.5)	2.7 (1.7, 5.0)	0.079
IL-4 pg/mL	70	8.6 (1.1, 26.0)	39.5 (4.5, 56.5)	0.009
IL-6 pg/mL	70	12.1 (3.9, 27.6)	14.5 (7.6, 44.0)	0.598
IL-10 pg/mL	70	12.4 (8.4, 15.7)	10.8 (7.1, 13.7)	0.092

The participants who had PTB culture-positive AFB microscopy smears negative had significantly higher CD4 and CD19 counts compared to those who were PTB culture-negative AFB microscopy smear negative. However, CD8 and CD16/56 were higher but not significant in PTB culture-positive AFB microscopy smears negative compared to PTB culture-negative AFB microscopy smears negative. Statistical significance was determined by two-sample Wilcoxon rank sum test.

Among AFB microscopy smear negative the study participants who were PTB culture-positive had significantly higher IFN-γ, TNF-α, and IL-2 profiles compared to those were PTB culture-negative (Table 2). Though there was no statistically significant difference in IL-12p70 between the PTB culture-positive AFB microscopy smears negative and culture-negative AFB microscopy smears negative. Statistical significance was determined by two-sample Wilcoxon rank sum test. The study participants who were culture-pusitive AFB microscopy smear negative had a significantly higher IL-4 profile compared to those who were culture-negative AFB microscopy smear negative, (*P* = *0*.*009*). Statistical significance was determined by two-sample Wilcoxon rank sum test.

The study further investigated the relationship between Th1 cytokines in PTB culture-negative and -positive AFB microscopy smear negative status by performing a two-sample Wilcoxon rank sum test. The data presented here demonstrate that Th1 cytokines IFN-γ (*P* < 0.001), TNF-α (*P* = 0.004) and IL-2 (*P* = 0.004) were elevated in PTB culture-positive AFB microscopy smear negative as compared to PTB culture-negative AFB microscopy smear negative. However, IL-12p70 (*P* = 0.079) was marginally elevated in PTB culture-positive AFB microscopy smear negative. The median level for IL-8 (*P* = 0.174) was comparable between the two groups (Table 2).

In order to explore the relationship between Th2 cytokines and PTB, we performed two-sample Wilcoxon rank sum test. The results revealed that there was an elevated IL-4 levels (*P* = 0.009) in PTB culture-positive AFB microscopy smear negative compared to PTB culture-negative AFB microscopy smear negative. However, the median levels of IL-6 (*P* = 0.598) and IL-10 (*P* = 0.092) were comparable between the two groups (Table 2).

### Comparisons of the PTB culture-negative and -positive AFB Microscopy smear negative to AFB microscopy smear positive

In order to determine the relationship between PTB culture-negative and -positive AFB microscopy smear negative to AFB microscopy smear positive a two sample Wilcoxon rank-sum test was performed and adjustment of the p-value for multiple comparison was done using Bonferroni method. Statistical significance was assessed at *P* < 0.0170. The median levels of CD8, CD4, CD16/CD56 and CD19 were comparable in all the three groups (Table 3).

**Table 3:** Comparison of culture-negative AFB microscopy smear negative, and culture-positive AFB microscopy smear negative to AFB smear positive.

Variable	TB Status		
	Culture Negative	Culture Positive	Sputum Positive
	N = 46	N = 24	N = 16
***Demographic Characteristics***			
Age (Years), Mean (SD)	41.2 (12.0)	34.3 (11.8)	40.0 (11.1)
Gender, n (%)			
Male	21 (45.7%)	14 (58.3%)	7 (43.8%)
Female	25 (54.3%)	10 (41.7%)	9 (56.2%)
***Immunophenotypic characteristics***			
CD8 (cells/µL), Median (IQR)	473.0 (157.8, 1166.0)	582.5 (217.5, 945.5)	466.5 (227.0, 668.2)
CD4 (cells/µL), Median (IQR)	67.5 (22.5, 192.5)	206.5 (101.0, 315.5)	233.5 (93.5, 262.2)
CD16/CD56 (cells/µL), Median (IQR)	63.0 (33.3, 113.5)	107.5 (56.6, 152.3)	55.0 (28.8, 128.0)
CD19 (cells/µL), Median (IQR)	46.0 (15.5, 99.3)	96.5 (29.5, 143.3)	26.5 (6.0, 44.0)
***Th1 Cytokines***			
IFN-γ (pg/mL), Median (IQR)	^*^7.6 (5.6, 16.9)	70.6 (8.6, 202.7)	56.0 (38.0, 133.8)
TNF-α (pg/mL), Median (IQR)	^*^16.1 (14.4, 19.2)	19.7 (15.8, 22.9)	21.2 (17.3, 29.7)
IL-2 (pg/mL), Median (IQR)	^*^15.3 (8.4, 26.9)	53.1 (14.1, 122.1)	73.2 (43.5, 164.5)
IL-8 (pg/mL), Median (IQR)	154.2 (50.1, 204.5)	118.1 (73.8, 158.6)	137.3 (80.4, 218.8)
IL-12p70 (pg/mL), Median (IQR)	^*^2.1 (0.7, 3.5)	2.7 (1.7, 5.0)	3.2 (2.1, 5.0)
***Th2 Cytokines***			
IL-4 (pg/mL), Median (IQR)	8.6 (1.1, 26.0)	^*^39.5 (4.5, 56.5)	9.4 (2.0, 19.6)
IL-6 (pg/mL), Median (IQR)	12.1 (3.9, 27.6)	14.5 (7.6, 44.0)	6.2 (3.5, 28.6)
IL-10 (pg/mL), Median (IQR)	12.4 (8.4, 15.7)	10.8 (7.1, 13.7)	10.0 (8.9, 13.2)

*Significantly different from the AFB smear positive participants at p-value < 0.0170 (Bonferroni corrected *P*-value for multiple comparisons).

Mean estimates were compared using independent samples t-test, and median estimates were compared using two sample Wilcoxon rank-sum test. The proportion of male participants in each group was compared using Pearson’s Chi Square test. There was no statistical difference in CD8, CD4, CD16/CD56 and CD19 immunophenotypic characteristics in study participants who were culture-negative AFB microscopy smear negative, and the culture-positive AFB smear negative to AFB microscopy smear positive.

The median levels of Th1 cytokines IFN –γ 56.0 pg/mL (38.0, 133.8) vs 7.6 pg/mL (5.6,16.9), TNF-α 21.2 pg/mL 17.3, 29.7) vs 16.1 pg/mL (14.4, 19.2), IL-2 73.2 pg/mL (43.5, 164.5) vs. 15.3 pg/mL (8.4, 26.9), IL-12p70 3.2 pg/mL (0.7, 3.5) vs. 2.1 pg/mL (0.7, 3.5) were elevated in AFB microscopy smear positive compared to PTB culture-negative AFB microscopy smear negative, but were comparable between PTB culture-positive AFB microscopy smear negative and AFB microscopy smear positive.

The data revealed that the median levels of IL-4, 39.5 pg/mL (4.5, 56.5) vs. 9.4 (2.0, 19.6) was elevated in PTB culture-positive AFB microscopy smear negative compared to AFB smear positive, but was comparable between PTB culture-negative AFB microscopy smear negative and AFB microscopy smear positive groups. The remaining median levels of Th2 cytokines were comparable among PTB culture-positive and -negative AFB microscopy smear negative, and AFB microscopy smear positive groups (Table 3).

### Relationship of Th1 and Th2 Cytokines levels and PTB culture-negative and -positive AFB microscopy smear negative study participants stratified by CD4 and CD8 levels

In order to investigate the effect of CD4 and CD8 on Th1 and Th2 cytokine levels among PTB culture-negative and -positive AFB microscopy smear negative, study participants were stratified by CD4 and CD8 levels and a two-sample Wilcoxon rank-sum test was performed and adjustment of the p-value for multiple comparison was done using Bonferroni method. Statistical significance was assessed at *P* < 0.0127. For the study participants with CD4 ≤ 200, the levels of Th1 cytokines [IFN-γ (*P* = 0.062), TNF-α (*P* = 0.579), IL-2 (*P* = 0.362), IL-8 (*P* = 0.756) and IL-12p70 (*P* = 0.460) and Th2 cytokines [IL-4 (*P* = 0.663), and IL-6 (*P* = 0.579) were comparable between PTB culture-negative and -positive AFB microscopy (both smear negative). However, IL-10 *(P* = *0*.*027)*] were elevated in culture-negative AFB microscopy (smear negative) as compared to culture-positive AFB microscopy (smear negative).

However, the data presented for the study participant with CD4 > 200 demonstrated elevated median Th1 cytokine levels IFN-γ 11.2 pg/mL (6.4,15.4) vs. 164.0 pg/mL (38.8, 240.8), *P* = 0.002, TNF–α 14.8 pg/mL (13.5, 16.5) vs. 22.2 pg/mL (18.9, 28.3), *P* < 0.001, IL-2pg/mL 13.7 (10.7, 37.3) vs 80.9 pg/mL (24.9, 153.3), *P* = 0.015 and IL-12p70 1.2 pg/mL (0.5, 1.9) vs. 3.8 pg/mL (2.3, 5.0), *P* = 0.001 in PTB culture-positive AFB microscopy (smear negative) as compared to PTB culture-negative AFB microscopy (smear negative). Only IL-4 median levels, 1.8 pg/mL (0.0, 13.0) vs. 54.3 (22.6, 71.2) *P* = 0.001, were higher in PTB culture-positive AFB microscopy (smear negative) as compared to PTB culture-negative AFB microscopy (smear negative) (Table 4).

**Table 4:** Comparison of Th1 and Th2 cytokines between culture negative AFB microscopy smear negative and culture positive AFB smear negative participants stratified by CD4 and CD8 levels.

Strata	Cytokines (pg/mL)	Culture negative AFB microscopy smear negative	Culture Positive AFB microscopy smear negative	*P*-value
		Median (IQR)		
CD4 ≤ 200	Th1 cytokines	N = 34	N = 11	
	IFN-γ	6.8 (5.6, 17.8)	23.6 (7.6, 92.8)	0.062
	TNF-α	17.1 (14.8, 19.7)	15.2 (15.0, 18.7)	0.579
	IL-2	15.9 (7.1, 25.7)	31.2 (5.0, 56.6)	0.362
	IL-8	127.5 (47.1, 202.5)^₮^	119.4 (112.7, 149.0)^§^	0.756
	IL-12p70	2.5 (1.4, 3.6)^₮^	1.3 (0.6, 3.2)^§^	0.460
CD4 > 200		N = 12	N = 13	
	IFN-γ	11.2 (6.4, 15.4)	164.0 (38.8, 240.8)	0.002
	TNF-α	14.8 (13.5, 16.5)	22.2 (18.9, 28.3)	<0.001
	IL-2	13.7 (10.7, 37.3)	80.9 (24.9, 153.3)	0.015
	IL-8	197.8 (144.0, 204.9)	135.2 (21.3, 168.4)	0.077
	IL-12p70	1.2 (0.5, 1.9)	3.8 (2.3, 5.0)	0.001
CD4 ≤ 200	Th2 cytokines	N = 34	N = 11	
	IL-4	10.7 (3.2, 28.7)	4.1 (1.8, 54.0)	0.663
	IL-6	14.9 (6.1, 52.4)	10.9 (6.4, 35.4)	0.579
	IL-10	13.4 (10.0, 19.8)	8.1 (7.0, 13.7)	0.027
CD4 > 200		N = 12	N = 13	
	IL-4	1.8 (0.0, 13.0)	54.3 (22.6, 71.2)	0.001
	IL-6	7.5 (3.1, 11.9)	16.9 (7.7, 42.1)	0.092
	IL-10	8.1 (7.6. 10.5)	11.6 (8.7, 13.3)	0.276
CD8 ≤ 1000	Th1 cytokines	N = 31	N = 19	
	IFN-γ	8.4 (5.0, 26.4)	52.0 (16.2, 170.0)	0.001
	TNF-α	16.5 (14.6, 18.5)	21.4 (16.2, 25.5)	0.002
	IL-2	12.3 (6.2, 25.1)	50.5 (19.8, 77.9)	0.003
	IL-8	154.0 (50.6, 206.1)^†^	119.4 (72.5, 151.5)^ħ^	0.406
	IL-12p70	1.9 (0.7, 3.4)^†^	2.3 (1.2, 5.6)^ħ^	0.161
CD8 > 1000		N = 15	N = 5	
	IFN-γ	6.8 (6.0, 11.2)	200.8 (5.2, 240.8)	0.457
	TNF-α	14.8 (14.2, 19.7)	18.1 (14.0, 18.2)	0.896
	IL-2	16.6 (11.5, 28.9)	157.3 (0.5, 158.8)	0.541
	IL-8	194.7 (39.9, 202.4)	164.9 (111.1, 168.4)	0.727
	IL-12p70	2.3 (1.1, 3.3)	3.4 (3.4, 3.8)	0.088
CD8 ≤ 1000	Th2 cytokines	N = 31	N = 19	
	IL-4	8.4 (0.2, 21.0)	38.4 (8.1, 57.4)	0.026
	IL-6	8.7(2.8, 19.3)	9.2 (5.5, 52.5)	0.401
	IL-10	12.7 (8.4, 20.7)	11.6 (7.1, 15.3)	0.159
CD8 > 1000		N = 15	N = 5	
	IL-4	8.9 (1.8, 27.9)	54.3 (2.6, 55.9)	0.221
	IL-6	22.0 (8.3, 50.1)	15.6 (14.9, 16.9)	0.861
	IL-10	10.4 (8.2, 14.9)	8.7 (7.2, 10.7)	0.238

^₮^N = 32; ^§^N = 10; ^†^N = 29; ^ħ^N = 18, p-value < 0.0127 (Bonferroni corrected P-value for multiple comparisons).

When we further stratified the study participants into those with CD4 ≤ 200 and CD4 > 200 and compared to culture-negative AFB microscopy smear negative and culture-positive AFB microscopy smear negative. There was no significant difference in levels of Th1 cytokines (IFN-γ, TNF-α, IL-2, IL-8 and IL-12p70) and Th2 cytokines (IL-4, IL-6 and IL-10) in study participants with CD4 ≤ 200.

However, in the study participants with CD4 > 200, there was significant difference in Th1 cytokines between culture-negative AFB microscopy smear negative and culture-positive AFB microscopy smear negative, IFN-γ (*P* = 0.002), TNF-α (*P* < 0.001), IL-2 (*P* = 0.015), and IL-12p70, *P* = 0.001). Only IL-4 median levels were significantly different (*P* = 0.001) between culture-negative AFB microscopy smear negative and culture-positive AFB microscopy smear negative among the study participants with CD4 > 200.

We further stratified the study participants into those with CD8 ≤ 1000 and CD8 > 1000 and compared to PTB culture-negative and -positive AFB microscopy smear negative. Among study participants who had CD8 ≤ 1000, IFN-γ (*P* = 0.001), TNF-α (*P* = 0.002), IL-2 (*P* = 0.003), and IL-4 (*P* = 0.026) were significantly different between culture-negative AFB microscopy smear negative and culture-positive AFB microscopy smear negative. While in study participants with CD8 > 1000, there was no difference in Th1 and Th2 cytokines levels evaluated between PTB culture-negative and -positive AFB microscopy smear negative.

We further investigated the interaction between CD4 or CD8 and the TB status in relationship to cytokines levels within each group [culture-positive AFB microscopy (smear negative) and culture -negative AFB (smear negative)] by performing a two sample Wilcoxon rank-sum test. The data demonstrated that median Th1 and Th2 cytokine levels were comparable within each respective group (Supplementary Table S1).

### Correlation among Th1 and Th2 Cytokine Levels and between Th1 and Th2 cytokines levels

The correlation among Th1 cytokines levels data demonstrated a concurrent increase in the median levels of IFN-γ and IL-2 cytokines (r = 0.48, *P* < *0*.*001*, power = 99.1%) and of IL-2 and TNF-α (r = 0.27, p = 0.22, power = 62.6%) indicating a synergistic relationship between the two cytokines when analyzed by Pearson correlation. On the contrary, it was evident that while median levels of TNF-α increased, that of IL-8 cytokine reduced (r = −0.41, p = 0.022, power = 93.9%) indicating an antagonistic relationship between the two cytokines.

Similarly, among the Th2 cytokines, the data indicated an increase in both the median levels of IL-4 and IL-10 (r = 0.29, *P* = 0.015, power = 69.1%) cytokines indicating a positive correlation between the two cytokines (Table 5).

**Table 5:** Correlation among Th1 and Th2 Cytokine Levels and between Th1 and Th2 cytokines levels.

	Pearson Correlation coefficient (r) (P-value)							
	INF-γ (pg/mL)	TNF-α (pg/mL)	IL2 (pg/mL)	IL8* (pg/mL)	IL12* (pg /mL)	IL4 (pg/mL)	IL6 (pg/mL)	IL10 (pg/mL)
**INF**-γ (pg/mL)	1.00							
**TNF**-α (pg/mL)	0.16 (0.178)	1.00						
**IL2** (pg/mL)	**0**.**48 (<0**.**001)**	**0**.**27 (0**.**022)**	1.00					
IL8* (pg/mL)	−0.10 (0.405)	**−0**.**41 (<0**.**001)**	−0.08 (0.513)	1.00				
IL12* (pg/mL)	−0.06 (0.634)	0.17 (0.173)	0.05 (0.701)	−0.16 (0.205)	1.00			
**IL4** (pg/mL)	−0.02 (0.880)	0.08 (0.513)	0.04 (0.752)	−0.07 (0.589)	−0.09 (0.487)	1.00		
**IL6** (pg/mL)	−0.10 (0.456)	**0**.**68 (<0**.**001)**	0.14 (0.208)	**−0**.**44 (<0**.**001)**	0.05 (0.701)	−0.09 (0.480)	1.00	
**IL10** (pg/mL)	−0.19 (0.119)	0.11 (0.346)	−0.12 (0.357)	0.23 (0.058)	0.01 (0.920)	**0**.**29 (0**.**015)**	0.03 (0.746)	1.00

*n = 67.

There was positive correlation between INF-γ and IL2 (r = 0.45, p < 0.001, power = 99.3%) demonstrating a synergistic relationship; an increase in the secretion of one of the cytokines was associated with an increase in the secretion of the other cytokine. Similarly, there was evidence of a moderate negative correlation between TNF - α and IL8 (r = −0.45, *P* < 0.001, power = 99.3%) indicating an antagonistic relationship; where an increase in the secretion of one of the cytokines was associated with a decrease in the secretion of the other cytokine, while the rest of the relationships between the Th1 cytokines were comparable (*P* > 0.05). Among the Th2 cytokines, there was a positive relationship between IL-4 and IL-10 (r = 0.28, *P* = 0.008, power = 75.0%), although not strong, an increase in the secretion of one of the two Th2 cytokines was associated with an increase in the other.

In order to assess the predictors of positive culture results among the AFB smear negative participants, binary logistic regression model was used. The inclusion of Th1 and the Th2 cytokines in the models was done in two steps. First, bivariate analyses were performed to assess the relationship between each of the cytokines on the culture positive test result. If the relationship was statistically significant the variable was included in the multivariable logistic regression model. The variables in the logistic regression model were assessed for multi- collinearity using the variance inflation factor (VIF) approach. The variables in the models had VIF that were less than 2, an evidence of lack of multi-collinearity.

The results presented provide evidence of a synergistic relationship between Th2 cytokine, IL-6 and Th1 cytokine, TNF-α (r = 0.68, *P* < 0.001, power = 97.3%), as an increase in one cytokine resulted in a concomitant increase in the other cytokine.

However, there was an inverse relationship between Th2 cytokine, IL-6 and Th1 cytokine, IL-8 (r = −0.44, *P* < 0.001, power >99.9), since an increase in IL-6 resulted in a decrease of IL-8 cytokine (Table 5).

### Predictive power of Th1 cytokines on PTB culture status among those who screened negative for AFB microscopy smears

**Figure 1:**
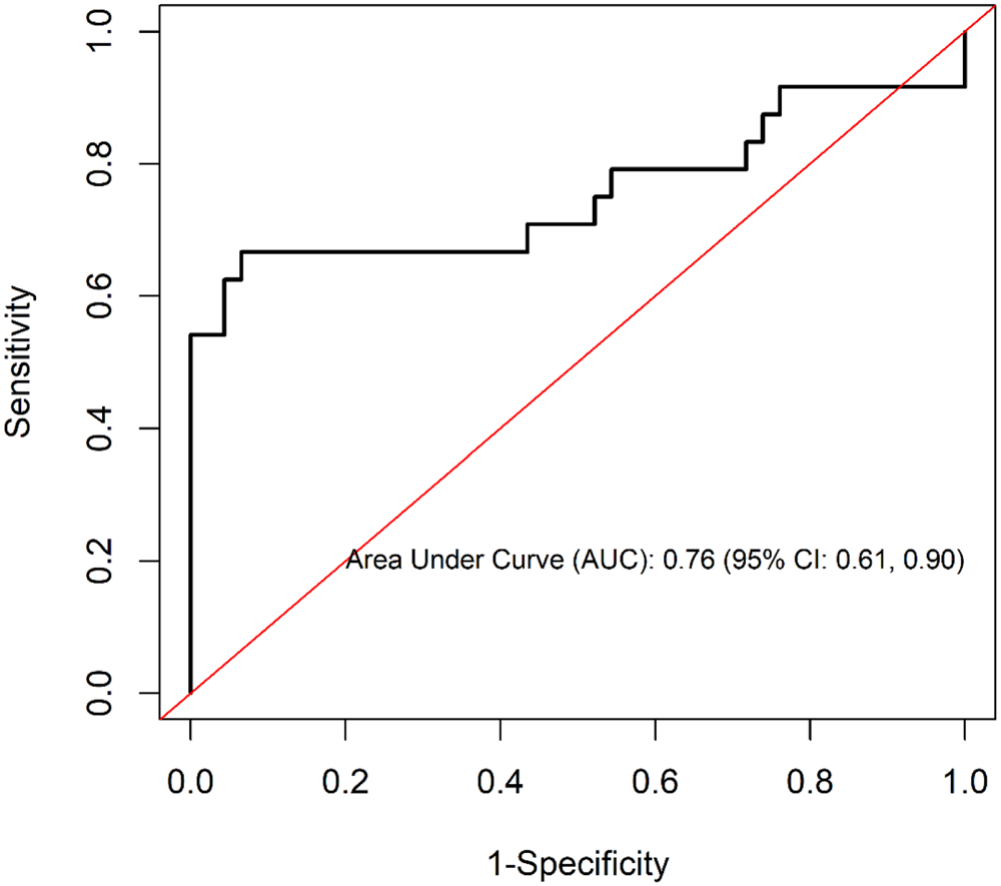
Predictive power of Th1 cytokines on PTB culture status among AFB microscopy smear negative. The model included INF-γ, TNF-α, IL2, CD4 (<200 vs. ≥200), and CD8 (<1000 vs. ≥1000).

To demonstrate the predictive power of Th1 cytokine on culture status, we performed a Receiver Operating Curve (ROC) analysis. The area under the curve (AUC) was 0.76 (95% CI: 0.61, 0.90) (Fig. 1), thus indicating a good predictive power of the ROC model for Th1 (IFN-γ, TNF-α, and IL-2), CD4 (<200 vs. ≥200), and CD8 (<1000 vs. ≥1000) covariates in discriminating between culture-positive and culture-negative in PTB patients who tested negative for AFB microscopy smears.

### Predictive power of Th2 cytokines on PTB culture status among those who screened negative for AFB microscopy smears

**Figure 2:**
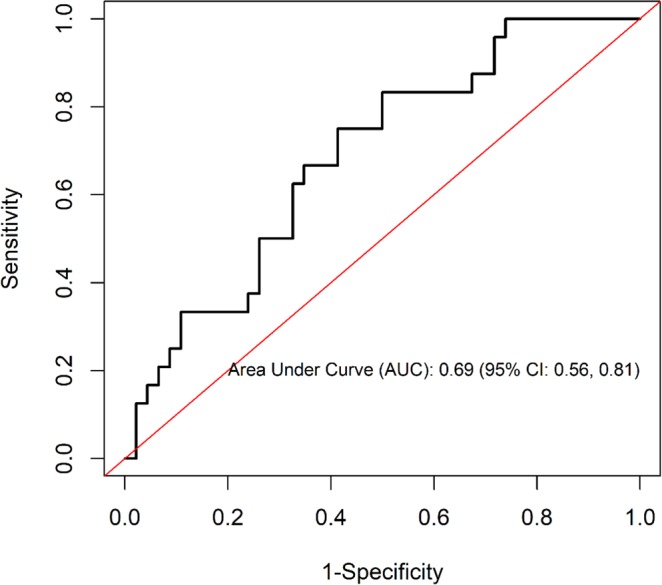
Predictive power of Th2 cytokines on PTB culture status among AFB microscopy smear negative. The model include IL4, IL6, IL10, CD4 (<200 vs. ≥200), and CD8 (<1000 vs. ≥ 1000).

In order to determine the predictive power of Th2 cytokines on PTB culture status among those who screened negative for AFB microscopy smears, a ROC analysis was performed. The area under the curve (AUC) was 0.69 (95% CI: 0.56, 0.81) (Fig. 2) implying that a randomly selected individual from the positive group has a test value larger than that for a randomly chosen individual from the negative group 69% percent of the times.

### Predictive power of Th1 cytokines, Th2 cytokines and T-cells (CD4, and CD8) on PTB culture status among AFB microscopy smear negative samples

**Figure 3:**
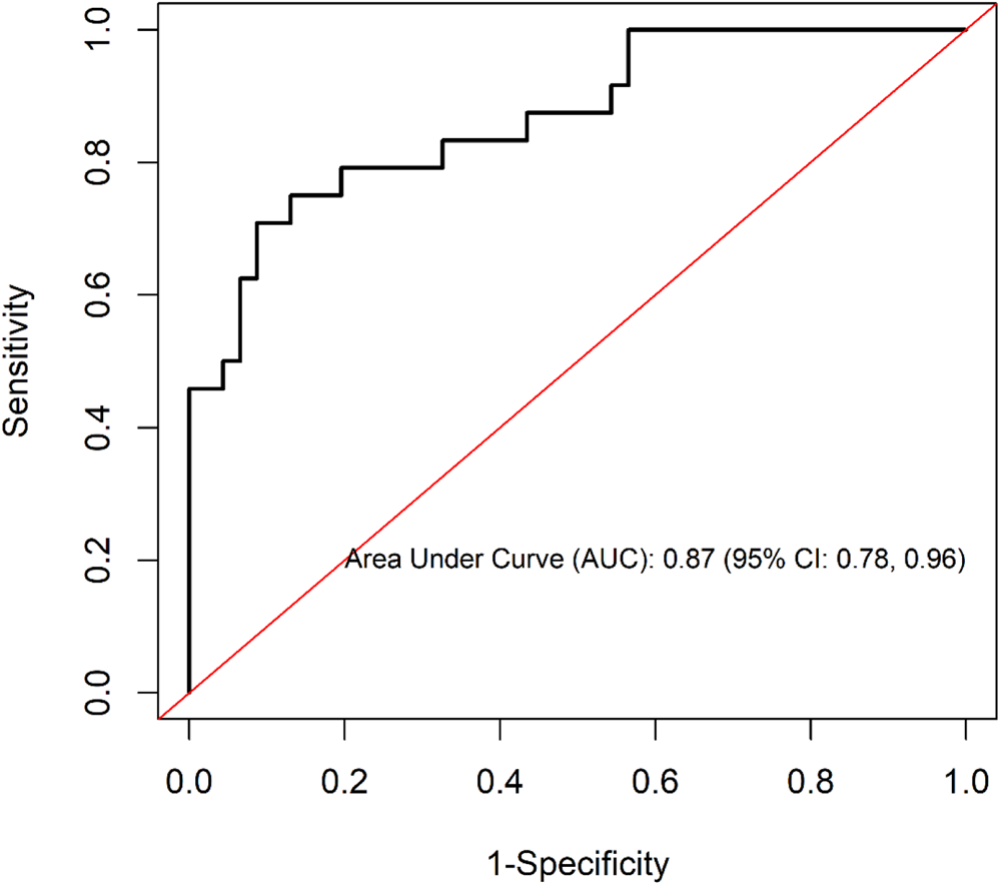
Predictive power of Th1 cytokines, Th2 cytokines and T-cells (CD4, and CD8) on PTB culture status among AFB microscopy smear negative. The model included IFN-γ, TNF-α, IL2, IL6, IL10, CD4 (<200 vs. ≥200), and CD8 (<1000 vs. ≥1000), but IL4 was eliminated due to inherent multicollinearity measured using variance inflation factor that was >5 units.

For us to demonstrate the predictive power of combined Th1 cytokines, Th2 cytokines and T cells (CD4 and CD8) among those who screened negative for AFB microscopy smears, a ROC analysis was performed. The predictive power of discriminating between those who were AFB smear negative in the diagnosis of PTB was good with cross validated area under the curve (AUC) being 0.87 (95% CI: 0.78, 0.96) (Fig. 3).

## Discussion

The development of a rapid diagnostic test for AFB microscopy (smear negative) pulmonary tuberculosis, and the ability to discriminate between the PTB culture-positive (AFB microscopy-smear negative) from PTB culture-negative (AFB microscopy-smear negative) would be a major advancement in the management and treatment of PTB. In the current study, we have demonstrated that Th1/Th2 cytokines measured in QFT-GIT test supernatants (plasma) have the potential to discriminate between positive and negative AFB microscopy negative smears.

Our study reveals that among the AFB microscopy smear negative PTB individuals, the median levels of CD4 counts were significantly higher in those who were culture-positive as compared to culture-negative individuals. Similar results were reported of higher levels of CD4 counts in study participants with PTB culture positive as compared to PTB culture negative^16^. Therefore, the higher levels of CD4 T cells observed in culture-positive AFB microscopy smear negative study participants are possibly an indication of activation of immune responses in reaction to proteins of phagocytosed bacilli, which are presented as peptides associated with class II major histocompatibility complex. This argument is in line with previous studies that demonstrated that the sites of active HIV/TB coinfection are key in the contribution to systemic immune activation^17^.

Significantly elevated levels of CD19+ B cell in culture-positive AFB microscopy smears negative participants compared to culture-negative AFB microscopy smears negative participants were reported in this study. This finding is similar to that of other studies that demonstrated increased antibody levels in culture-positive as compared to culture-negative individuals when their peripheral blood were stimulated with TB-specific antigens^18,19^. Collectively, these underscores the important role of antibody-mediated immunity resulting from Class II MHC restricted T cell-B cell collaboration driven by antigen-receptor interactions, which cause the activation of both B and T cells.

In the present study, the levels of IFN-γ were significantly higher in culture-positive (AFB microscopy smear negative) as compared to culture-negative (AFB microscopy smear negative). This finding is in agreement with a previous study that indicated higher levels of IFN-γ in culture-positive as compared to culture-negatives patients’ peripheral blood stimulated with TB-specific antigens^20^. It is also important to note that IFN-γ levels were high in the study participants in that study irrespective of low or high CD4 counts. Taken together, these findings indicate that IFN-γ production in PTB patient may be as a result of activation of *Mycobacterium*-specific T cells. Moreover, elevated levels of IFN-γ in HIV-TB co-infection with significant raise in IFN-γ/IL-10 ratio have been known to alter the impact of IL-10 inhibitory function in HIV infection, thus leading to a shift towards a more dominant pro-inflammatory reaction (Th1), potentially resulting from *Mycobacterium tuberculosis*-induced pathogenesis^21^.

Similar to other studies carried out, we observed elevated levels of TNF-α in culture-positive (AFB microscopy smear negative) as compared to culture-negative (AFB microscopy smear negative) individuals. Higher levels of TNF-α have been reported in culture supernatants of peripheral blood in patients with PTB stimulated with mycobacterial antigens, suggesting a role with other cytokines such as IFN-γ in the control of *Mycobacterial tuberculi* multiplication^22,23^.

A previous study reported significantly increased levels of IL-2 in TB patients after stimulation with ESAT-6^24^. This was in line with findings from the current study that reported higher levels of IL-2 in culture-positive (AFB microscopy smear negative) as compared to culture-negative (AFB microscopy smear negative) individuals. Furthermore, it is has been reported that measurement of multiple cytokines would be important in determining PTB smear status especially among TB-HIV co-infection patients^25,26^. Similarly, it has been demonstrated in other studies that there are elevated levels of IL-2 and IFN-γ during active TB when the bacilli load is high, an observation which was supported by our observation in the current study^27^. The IFN-γ plays a critical role in the activation of macrophages and thus the control of *Mycobacterium tuberculosis* bacilli whereas IL-2 is known to stimulate the proliferation of T cell. Increased levels of these two cytokines in culture-positive (AFB microscopy smear negative), may demonstrate the presence of MTB infection through their involvement in initiation of immune response against the bacilli.

We further assessed the relationship between Th2 cytokines and AFB microscopy smears status. We noted significant differences in median levels of IL-4 in culture-positive (AFB microscopy smear negative) as compared to culture-negative (AFB microscopy smear negative). This corroborate the findings from a study that reported increased expression of IL-4 to be associated with the virulence factor, leading to the promotion of anti-inflammatory functions and the ability to cause tissue damage in conjunction with TNF-α^28^.

In the present study, we further evaluated if the low levels of Th1 and Th2 cytokines in AFB smear negative PTB-HIV co-infected study participants were because of depleted CD4 and CD8 T cells. We stratified our participants into categories of CD4 ≤ 200 cells/µl vs. CD4 > 200 cells/µl, and CD8 ≤ 1000 cells/µl vs. CD8 > 1000 cells/µl. In CD4 > 200 cells/µl category, there was significant difference in Th1 [IFN-γ, TNF-α, IL-2 and IL-12(p70)] and Th2 (IL-4) cytokines observed between culture-positive (AFB microscopy smear negative) and culture-negative (AFB microscopy smear negative) study participants. Similar findings were reported in another study carried out in India^29^. However, among our study participants with CD4 ≤ 200 cells/µl, there were no significant differences reported in Th1 and Th2 cytokines between the two groups, except IL-10. This was similar to previous findings^29^ that demonstrated that despite low CD4 ≤ 200 cells/µl HIV-TB patients had significantly elevated Th2 (IL-4 and IL-10) cytokines as compared to HIV patients. This may be attributed to the extent and interaction of the immune system in response to HIV and/or TB co-infection.

In regard to CD8, the data revealed significant difference in Th1 cytokines (IFN-γ, TNF-α, IL-2) and Th2 cytokine (IL-4) among study participants who had CD8 ≤ 1000 when comparing AFB smear negative culture negative and AFB smear negative culture positive. However, there was no difference observed in Th1 and Th2 cytokine levels between AFB smear negative culture negative and culture positive individuals with CD8 > 1000. The role of CD8 remains unclear, with some studies reporting that CD8 might have an important function in conferring immunity against TB through the secretion of cytolysin or perforins^30,31^, while others report the existence of CD8 might be harmful, since CD8 levels were elevated in AFB smear negative culture positive as compared to AFB smear negative culture negative individuals.

In the current study, the highest AUC after ROC analysis to differentiate between culture-positive AFB smear negative and culture-negative AFB smear negative was observed in combined cytokines measurements as compared to single cytokine measurements. Similar findings have been reported in other studies in which the use of either multiple cytokines measurements or cytokine ratios were proposed to be more useful in diagnosis of PTB. This was to cater for the inter-individual variability when only one cytokine is used, which ideally would make these immune-diagnostic tests unreliable especially in immune-compromised individuals^9,10^.

## Conclusion

In conclusion, we have identified a combination of multiple cytokines, both Th1 (IFN-γ, TNF-α & IL-2) and Th2 (IL-6, and Il-10) that could discriminate between culture-positive AFB smear negative and culture-negative AFB smear negative. This will go a long way in ensuring that this group of patients is correctly diagnosed for effective therapeutic interventions. It is also important to note that culture-negative AFB smear negative individual who have low production of cytokines in response to *Mycobacterium tuberculosis*-specific antigens might be infected potentially by another disease and not PTB. Most importantly, we propose that a blood cytokine signature could identify those who are culture-positive AFB smear negative from those who ultimately are culture-negative AFB smear negative and do not have PTB. The implication of this in the management of PTB is that attempts should be made to carry out blood tests rather than cultures so as to improve on the time required to make PTB treatment decisions, since the blood test could be done much quicker than culture. Lastly, the extension of this study to a larger cohort would be important in order to validate the diagnostic accuracy of both Th1 (IFN-γ, TNF-α & IL-2) and Th2 (IL-6, and Il-10) biomarkers.

## Data Availability

The study’s data, numerical and text after de-identification would be available upon written request from the senior author.

## Supplementary information

Supplementary Info
