# Supplementary material for: The diagnostic accuracy of Th1 (IFN-γ, TNF-α, and IL-2) and Th2 (IL-4, IL-6 and IL-10) cytokines response in AFB microscopy smear negative PTB- HIV co-infected patients
Source: Sci Rep. 2019 Feb 27;9:2966. doi: 10.1038/s41598-019-39048-x (PMC6393479; doi:10.1038/s41598-019-39048-x)
Supplement: Supplementary file 1 — Supplementary Info [file 41598_2019_39048_MOESM1_ESM.docx]

**The diagnostic accuracy of Th1 (IFN-γ, TNF-α, and IL-2) and Th2 (IL-4, IL-6 and IL-10) cytokines response in AFB microscopy smear negative PTB- HIV co-infected patients**

Job Kisuya^1, 2*^, Alex Chemtai^3^, Evans Raballah^4, 7^, Alfred Keter^2^, Collins Ouma^1, 5, 6^

1. Department of Biomedical Science and Technology, Maseno University, Private Bag, Maseno, Kenya
2. Academic Model for Providing Access to Healthcare (AMPATH), P.O Box 4606-30100 Eldoret, Kenya
3. Department of Immunology, Moi University, P.O. Box 4606-30100, Eldoret, Kenya
4. Department of Medical Laboratory Sciences, Masinde Muliro University of Science and Technology, P.O. Box 190-50100, Kakamega, Kenya
5. Centre for Global Health Research/Kenya Medical Research Institute, P.O. Box 1578-40100, Kisumu, Kenya
6. Ideal Research Centre, P.O. Box 7244-40123, Kisumu, Kenya
7. Center for Global Health, Department of Internal Medicine, University of New Mexico Health Sciences Center, Albuquerque, NM, USA

**Supplementary Table S1: Comparison of Th1 & Th2 cytokine levels by CD4 and CD8 levels (or groups) within the PTB culture-negative and -positive AFB microscopy smear negative.**

|  | Culture Negative AFB microscopy smear -ve | |  | Culture Positive AFB microscopy smear -ve | |  |
| --- | --- | --- | --- | --- | --- | --- |
|  | CD4 ≤ 200 | CD4 > 200 |  | CD4 ≤ 200 | CD4 > 200 |  |
| Cytokines (pg/mL) | N = 34 | N = 12 | *P*-value | N = 11 | N = 13 | P-value |
|  | Median (IQR) | |  | Median (IQR) | |  |
| Th1 cytokines |  |  |  |  |  |  |
| IFN -γ | 6.8 (5.6, 17.8) | 11.2 (6.4, 15.4) | 0.539 | 23.6 (7.6, 92.8) | 164.6 (38.8, 240.8) | 0.077 |
| TNF - α | 17.1 (14.8, 19.7) | 14.8 (13.5, 16.5) | 0.048 | 15.2 (15.0, 18.7) | 22.2 (18.9, 28.3) | 0.003 |
| IL-2 | 15.9 (7.1, 25.7) | 13.7 (10.7, 37.3) | 0.476 | 31.2 (5.0, 56.6) | 80.9 (24.9, 153.3) | 0.056 |
| IL-8 | 127.5 (47.1, 202.5)^₮^ | 197.8 (144.0, 204.9) | 0.192 | 119.4 (112.7, 149.0)^§^ | 135.2 (21.3, 168.4) | 0.687 |
| IL-12p70 | 2.5 (1.4, 3.6)^₮^ | 1.2 (0.5, 1.9) | 0.051 | 1.3 (0.6, 3.2) ^§^ | 3.8 (2.3, 5.0) | 0.077 |
| Th2 cytokines |  |  |  |  |  |  |
| IL-4 | 10.7 (3.2, 28.7) | 1.8 (0.0, 13.0) | 0.110 | 4.1 (1.8, 54.0) | 54.3 (22.6, 71.2) | 0.020 |
| IL-6 | 14.9 (6.1, 52.4) | 7.5 (3.1, 11.9) | 0.107 | 10.9 (6.4, 35.4) | 16.9 (7.7, 42.1) | 0.664 |
| IL-10 | 13.4 (10.0, 19.8) | 8.1 (7.6, 10.5) | 0.016 | 8.1 (7.0, 13.7) | 11.6 (8.7, 13.3) | 0.505 |
|  | CD8 ≤ 1000 | CD8 > 1000 |  | CD8 ≤ 1000 | CD8 > 1000 |  |
| Cytokines (pg/mL) | N = 31 | N = 15 | P-value | N = 19 | N = 5 | P-value |
|  | Median (IQR) | |  | Median (IQR) | |  |
| Th1 cytokines |  |  |  |  |  |  |
| IFN -γ | 8.4 (5.0, 26.4) | 6.8 (6.0, 11.2) | 0.681 | 52.0 (16.2, 170.0) | 200.8 (5.2, 240.8) | 0.803 |
| TNF - α | 16.5 (14.6, 18.5) | 14.8 (14.2, 19.7) | 0.716 | 21.4 (16.2, 25.5) | 18.1 (14.0, 18.2) | 0.046 |
| IL-2 | 12.3 (6.2, 25.1) | 16.6 (11.5, 28.9) | 0.170 | 50.5 (19.8, 77.9) | 157.3 (0.5, 158.8) | 0.670 |
| IL-8 | 154.0 (50.6, 206.1)^†^ | 194.7 (39.9, 202.4) | 0.980 | 119.1 (72.5, 151.5) ^ħ^ | 164.9 (111.1, 168.4) | 0.4343 |
| IL-12p70 | 1.9 (0.7, 3.4) ^†^ | 2.3 (1.1, 3.3) | 0.519 | 2.3 (1.2, 5.6) ^ħ^ | 3.4 (3.4, 3.8) | 0.502 |
| Th2 cytokines |  |  |  |  |  |  |
| IL-4 | 8.4 (0.2, 21.0) | 8.9 (1.8, 27.9) | 0.805 | 38.4 (8.1, 57.4) | 54.3 (2.6, 55.9) | 0.831 |
| IL-6 | 8.7 (2.8, 19.3) | 22.0 (8.3, 50.1) | 0.046 | 9.2 (5.5, 52.5) | 15.6 (14.9, 16.9) | 0.619 |
| IL-10 | 12.7 (8.4, 20.7) | 10.4 (8.2, 14.9) | 0.512 | 11.6 (7.1, 15.3) | 8.7 (7.2, 10.7) | 0.355 |

^₮^ N = 32; ^§^ N = 10; ^†^ N = 29; ^ħ^ N = 18

The top left panel of Supplementary Table S1 presents the comparison of Th1 & Th2 cytokine levels between the participants with CD4 ≤ 200 cells/µl and those with CD4 > 200 cells/µl among those who were culture-negative AFB microscopy smear negative. The findings do not demonstrate strong evidence of a difference in the cytokine levels by the CD4 levels. Similarly, the top right panel of Table S1 presents the comparison of Th1 & Th2 cytokine levels between the participants with CD4 ≤ 200 cells/µl and those with CD4 > 200 cells/µl among those who were culture-positive AFB microscopy smear negative.

Similar comparisons of cytokine levels by CD8 levels was done among those who were culture-negative AFB microscopy smear negative (bottom left panel of Supplementary Table S1), and culture-positive AFB microscopy smear negative (bottom right panel of Supplementary Table S1). There was no strong evidence from the findings to suggest an effect of CD8 levels on the cytokine levels within the specific culture status among the AFB smear negative participants.

The lack of difference in Supplementary Table S1 is a clear statistical indication of lack of interaction between CD4 or CD8 and the TB status to influence the levels of the cytokines.
